# Neuroprotective Potentials of Panax Ginseng Against Alzheimer’s Disease: A Review of Preclinical and Clinical Evidences

**DOI:** 10.3389/fphar.2021.688490

**Published:** 2021-06-02

**Authors:** Jing Li, Qingxia Huang, Jinjin Chen, Hongyu Qi, Jiaqi Liu, Zhaoqiang Chen, Daqing Zhao, Zeyu Wang, Xiangyan Li

**Affiliations:** ^1^ Jilin Ginseng Academy, Key Laboratory of Active Substances and Biological Mechanisms of Ginseng Efficacy, Ministry of Education, Jilin Provincial Key Laboratory of Bio-Macromolecules of Chinese Medicine, Changchun University of Chinese Medicine, Changchun, China; ^2^ Research Center of Traditional Chinese Medicine, College of Traditional Chinese Medicine, Changchun University of Chinese Medicine, Changchun, China; ^3^ Department of Scientific Research, Changchun University of Chinese Medicine, Changchun, China

**Keywords:** Alzeheimer’s disease, ginseng (*Panax ginseng* C.A. Meyer), ginsenosidase, gintonin, neuroprotection

## Abstract

Alzheimer’s disease (AD), a neurodegenerative disorder, is a major health concern in the increasingly aged population worldwide. Currently, no clinically effective drug can halt the progression of AD. *Panax ginseng* C.A. Mey*.* is a well-known medicinal plant that contains ginsenosides, gintonin, and other components and has neuroprotective effects against a series of pathological cascades in AD, including beta-amyloid formation, neuroinflammation, oxidative stress, and mitochondrial dysfunction. In this review, we summarize the effects and mechanisms of these major components and formulas containing *P. ginseng* in neuronal cells and animal models. Moreover, clinical findings regarding the prevention and treatment of AD with *P. ginseng* or its formulas are discussed. This review can provide new insights into the possible use of ginseng in the prevention and treatment of AD.

## Introduction

Alzheimer’s disease (AD) is a neurodegenerative disorder and is one of the most common causes of dementia in the elderly population (Jia et al., 2020). The global costs in 2030 due to dementia could be much higher than the predictions made by the World Alzheimer Report 2015, reaching $2.54 trillion (Jia et al., 2018). According to the Alzheimer’s Association, the incidence and prevalence, mortality and morbidity, use and costs of care, and the overall impact on the caregivers and society of AD are increasingly major concerns (Alzheimer’s Association, 2020). The prevention and treatment of AD has become a global problem due to the increasingly aged population worldwide (Alzheimer’s Association, 2020; Ikram et al., 2020). AD clinically manifests as apathy, anxiety, cognitive and functional decline, and the emergence of neuropsychiatric symptoms (Johansson et al., 2020; Cummings, 2021).

AD pathogenesis is defined by the extracellular deposition of beta-amyloid (Aβ) and tau hyperphosphorylation (Vaz and Silvestre, 2020). Aβ plaque formation is thought to be the main cause of AD symptoms, including memory deficit, due to its neurotoxic effect (Yankner et al., 1989; Sonawane et al., 2021). Aβ is derived from Aβ protein precursors (AβPPs) through the amyloidogenic pathway (Hwang et al., 2012; Kwon et al., 2019). Aβ accumulation accelerates tau phosphorylation (p-tau) during AD development (Gomes et al., 2019), whereas normal tau phosphorylation is essential for neuronal plasticity and axonal outgrowth (Arendt and Bullmann, 2013). Hyperphosphorylated tau protein is released from microtubules and self-assembles into neurotoxic insoluble aggregates such as intracellular neurofibrillary tangles (NFTs) (JeffKuret, 2008). The toxic effects of senile plaques composed of Aβ peptides and NFTs on the brain cholinergic system, mitochondria, and axonal transport result in oxidative stress, intracellular Ca^2+^ overload, apoptosis, and glutamate dysregulation (Bader Lange et al., 2008; Aalinkeel et al., 2018). In addition, the senile plaques produce more Aβ peptides through microglial activation and release of pro-inflammatory cytokines. The treatments for AD approved by the Food and Drug Administration are mainly based on reducing acetylcholine (ACh) levels and glutamate excitotoxicity and inhibiting Aβ protein deposition in the brain; approved drugs include donepezil, rivastigmine hydrogen tartrate, galanthamine, and huperzine-A (Li et al., 2019; Kareti and P, 2020; Pardridge, 2020). Although these drugs can result in symptomatic improvement, they cannot reverse AD progression and cause various adverse effects after long-term use.

*Panax ginseng* C.A. Mey*.* (ginseng) is a well-known and valuable medicinal herb that has been widely used in China and other East Asian countries as traditional Chinese medicine and health food (Shi et al., 2019). Recent studies demonstrated that ginseng extracts, active components (ginsenosides and gintonin), and ginseng formulas can improve the symptoms of AD patients and inhibit the progression of AD by reducing the deposition of Aβ and tau protein hyperphosphorylation. These effects may be mediated by mitochondrial function, neuron conduction, apoptosis, calcium ions, and reactive oxygen species (ROS) (Rajabian et al., 2019; Guo et al., 2021). Ginsenosides, which are mainly classified into protopanaxadiol (PPD) and protopanaxatriol (PPT), according to their sapogenin, can result in significant improvement in AD symptoms (Im and Nah, 2013; Kim et al., 2018; Piao et al., 2020). Previous studies have confirmed that β-site APP cleaving enzyme 1 (BACE1, β-secretase) inhibitors can inhibit the formation of Aβ (Karpagam et al., 2013), and acetylcholinesterase (AChE) inhibition can improve cognitive and memory function (Park et al., 2017). Molecular dynamics analysis combined with enzyme activity experiments showed that ginsenosides CK, F1, Rh1, and Rh2 are potential BACE1 inhibitors, inhibiting the formation of Aβ (Karpagam et al., 2013). In addition, ginsenosides F1, Rd, Rk3, 20(S)-Rg3, F2, and Rb2 possess strong AChE inhibitory activities, which can improve cognitive and memory function (Nah, 2012; Yang et al., 2019a). Gintonin, a component of ginseng, is a bioactive glycolipoprotein that forms nonsaponin multimers (Pyo et al., 2011; Nah, 2012; Jakaria et al., 2020; Choi et al., 2021). Recent studies have shown that gintonin can affect the activation of the phosphatidic acid receptor, which is involved in hemolysis, reducing the formation of Aβ and improving learning and memory abilities (Lee et al., 2018a; Kim et al., 2018). In addition, gintonin can also reduce the symptoms and progress of AD through neurogenesis, autophagy stimulation, anti-apoptosis effects, anti-oxidative stress, and anti-inflammatory activities (Choi et al., 2021).

We first introduce the effects and mechanisms of ginsenosides, gintonin, and ginseng formulas in the prevention and treatment of AD based on the extensive *in vitro* and *in vivo* studies. Then, we summarize the clinical findings regarding the prevention and treatment of AD with ginseng or its formulas. This review can provide new insights into the possible use of ginseng in AD treatment.

## Effects and Mechanisms of Ginseng in Preventing and Treating AD

### Ginsenosides

It has been reported that many ginsenosides can target the following pathological processes of AD: (1) inhibiting Aβ aggregation and tau hyperphosphorylation, (2) protecting against neuroinflammation and apoptosis, (3) increasing the secretion of neurotrophic factors, and (4) improving mitochondrial dysfunction.

#### Aβ Aggregation and Tau Hyperphosphorylation

In Aβ_1–40_-induced AD rats, ginsenoside Rb1 can improve learning and memory by altering the amyloidogenic process of APP into a nonamyloidogenic process (Lin et al., 2019). Ginsenoside Rb1, an agonist of peroxisome proliferator–activated receptor-γ (PPARγ), could lower cholesterol levels and reduce the cytotoxicity induced by Aβ_25–35_ by decreasing lipid peroxidation and protecting the rigidity of the cytoskeleton and the membrane surface in PC12 cells (Changhong et al., 2021). Ginsenoside Rd increases soluble APP-α (sAPPα) levels and reduces extracellular Aβ levels, enhancing cognitive and memory functions of ovariectomized rats (Yan et al., 2017). Ginsenoside Re inhibits the activity of BACE1 by increasing PPARγ expression at the mRNA and protein levels in N2a/APP695 cells and thereby reduces the generation of Aβ_1–40_ and Aβ_1–42_ (Cao et al., 2016). Another research showed that ginsenoside Rg1 can downregulate cyclin-dependent kinase 5 (CDK5) expression to inhibit the phosphorylation of PPARγ and the activity of its targets, BACE and insulin-degrading enzyme (IDE), reducing Aβ levels, and exerting neuroprotective effects against AD (Quan et al., 2020). In SweAPP-SK cells with mutant APP, Rg3 treatment significantly enhances neprilysin (NEP) gene expression, reducing the levels of Aβ_40_ and Aβ_42_ (Yang et al., 2009).

With respect to tau hyperphosphorylation, Rd pretreatment can maintain the functional balance between glycogen synthase kinase 3β (GSK-3β) and protein phosphatase 2A (PP-2A), inhibiting tau phosphorylation (Li et al., 2013). Moreover, Rd inhibits the hyperphosphorylation of tau protein at Ser199/202, Ser396, or Ser404, induced by okadaic acid microinfusion in rats and cortical neurons, increasing the PP-2A activity and protecting against AD (Li et al., 2011). Collectively, these results suggest that ginsenosides Rb1, Rd, Re, and Rg1 can inhibit Aβ aggregation to regulate the phosphorylation of tau protein in the prevention and treatment of AD.

#### Neuroinflammation

Neuroinflammation is a continuous process that is implicated in the preclinical, moderate, and late stages of AD (Sung et al., 2020). In APP transgenic mice, Rd pretreatment at 10 mg/kg significantly suppresses the NF-κB pathway activity, reducing the generation of pro-inflammatory cytokines, such as interleukin-1 beta (IL-1β), IL-6, tumor necrosis factor-α (TNF-α), and S100 calcium-binding protein B (S100β), which can improve learning and memory abilities (Liu et al., 2015a). Meanwhile, Rd exerts obvious anti-inflammatory, anti-oxidative, and anti-apoptotic effects by reducing caspase-3 expression and apoptosis of normal cells in Aβ_1–40_-induced AD model rats (Liu et al., 2012). Ginsenoside Rf significantly alleviates Aβ-induced neuronal death in N2A cells and memory deficits in Aβ-treated mice by alleviating inflammation and enhancing Aβ degradation, which suggests that Rf decreases Aβ-induced neurotoxicity during AD development (Du et al., 2018). Ginsenoside Rg1 can reduce the NADPH oxidase 2 (NOX2)–mediated ROS production and neuronal apoptosis, which in turn inhibits the nucleotide-binding domain and leucine-rich repeat pyrin domain–containing protein 1 (NLRP1) inflammasome in H_2_O_2_-induced hippocampal neurons (Xu et al., 2019). Moreover, the combination of Rb1 with Rg1 can reduce brain Aβ production by regulating multiple processes, including NLRP3 inflammasome, TNF-α levels, oxidative stress, and astrocyte and microglia activation (Yang et al., 2020). Rb1 has a stronger effect on reducing the levels of apoptosis-related proteins in the hippocampus, and Rg1 has a stronger effect in decreasing iNOS levels and activating glial cells (Yang et al., 2020). In addition, ginsenoside Rg1 suppresses the TLR3/4 signaling pathway to decrease inflammatory factors in Aβ_25–35_-induced NG108-15 cells (Zhao et al., 2014). In lipopolysaccharide (LPS)-induced rats, Rg3 administration significantly alleviates cognitive impairment by inhibiting the expression of pro-inflammatory mediators (TNF-α, IL-1β, and cyclooxygenase 2 [COX-2]) in the brain (Lee et al., 2013). In Aβ_42_-treated BV-2 cells, the binding of NF-κB p65 to its DNA consensus sequences and TNF-α expression in activated microglia are effectively reduced by Rg3 treatment (Joo et al., 2008). Compound K (CK), a metabolite biotransformed from ginsenosides Rb1, Rb2, and Rc (Oh and Kim, 2016), can suppress various inflammatory molecules in LPS-stimulated BV2 cells and primary microglia by regulating the mitogen-activated protein kinase (MAPKs), NF-κB/AP-1, and HO-1/ARE signaling pathways (Park et al., 2012). These *in vitro* and *in vivo* findings indicate that major ginsenosides can alleviate inflammation in hippocampal neurons and microglia by mainly regulating the NF-κB pathway and NLRP3 inflammasome.

#### Neurotrophic Factors

Neurotrophic factors are endogenous proteins that maintain survival and differentiated functions of neurons, including the brain-derived neurotrophic factor (BDNF) and tropomyosin-related kinases (Trks) A, B, and C (Schindowski et al., 2008). A study showed that Rb1 can promote endogenous neural stem cell proliferation and differentiation by increasing the protein levels of Nestin, glial fibrillary acidic protein (GFAP), and nucleotide sugar epimerase (NSE), thereby improving cognitive function of AD rats (Zhao et al., 2018). Ginsenoside F1 can decrease phosphorylated cAMP-response element binding protein (CREB) and increase cortical BDNF levels in the hippocampus, reducing Aβ plaques and improving memory function of APP/PS1 double-transgenic AD mice (Han et al., 2019). With respect to other neurotrophic factors, the gene and protein expression levels of the nerve growth factor receptor p75 and TrkA in Neuro2a cells are increased by ginsenoside Re and Rd, which suggest that the NGF-TrkA signaling pathway mediates the ginsenoside-induced neuroprotective effects against AD progression (Kim et al., 2014).

#### Apoptosis

The balance between of pro-apoptotic and anti-apoptotic factors in brain tissue plays important roles in improving cognitive and memory functions in AD. Rb1 administration significantly reduces the levels of Bax and cleaved caspase-3 and enhances Bcl-2 levels in the hippocampus to prevent cognitive deficit of Aβ_1–40_-induced rats (Wang et al., 2018; Lin et al., 2019). In Aβ_25–35_-induced PC12 cells and hippocampal CA1 neurons, Rg2 improves cell survival and inhibits apoptosis by promoting the Bcl-2/Bax ratio and attenuating the cleavage of caspase-3, which is mediated by the enhancement of PI3K/Akt signaling (Cui et al., 2017; Cui et al., 2020). Meanwhile, Aβ_25–35_-induced oxidative stress and neuronal apoptosis are, obviously, ameliorated by Rd by keeping the oxidation–anti-oxidation balance and regulating apoptotic proteins, such as Bax, Bcl-2, and cytochrome c (Cyto C) (Liu et al., 2015b). In Aβ-induced SH-SY5Y cells, Re can elevate the ratio of Bcl-2/Bax and reduce the release of Cyto C to maintain mitochondrial function by regulating the apoptosis signal–regulating kinase 1 (ASK1)/JNK/Bax and Nrf2/HO-1 pathways (Liu et al., 2019). Ginsenoside Rg2 significantly attenuates glutamate-induced neurotoxic effects through mechanisms related to anti-oxidative (malondialdehyde [MDA] and nitrogen oxide [NO]) and anti-apoptotic (caspase-3) mechanisms (Li et al., 2007). In a scopolamine-exposed AD mouse model, CK was found to enhance Nrf2/Keap1 signaling, increasing the anti-oxidative activity and reducing neuronal apoptosis, which can regulate the balance between Aβ production and clearance and improve memory function (Yang et al., 2019b). Taken together, ginsenosides Rb1, Rg2, Re, and Rd can regulate apoptosis-related proteins, including Bcl-2, Bax, and Cyto C, reducing Aβ-induced or tau protein–induced neurotoxicity during AD development.

#### Mitochondrial Dysfunction

Mitochondrial dysfunction, including mtDNA lesions and reduced electron transport chain (ETC) enzyme function, is found in the brains of AD subjects, highlighting potential treatment strategies for AD (Perez Ortiz and Swerdlow, 2019). Metabolomic analysis showed that Re treatment can restore metabolic profiling including lecithin, amino acids, and sphingolipids, to exert protective effects in AD mice (Li et al., 2018). Rg1 can improve memory impairment and depression-like behavior in 3 × Tg-AD mice by upregulating the expression of the depression and memory-related proteins complexin-2 (CPLX2), synapsin-2 (SYN2), and synaptosomal-associated protein 25 (SNP25) (Nie et al., 2017). In AD rats, Rg3 can prevent cognitive impairment by directly or indirectly improving mitochondrial dysfunction, ETC function, and amino acid/purine metabolism (Zhang et al., 2019). In Aβ-induced HT22 cells, CK treatment can regulate abnormal expression of proteins related to energy metabolism, promoting Aβ degradation and inhibiting tau expression (Chen et al., 2019).

**FIGURE 1:**
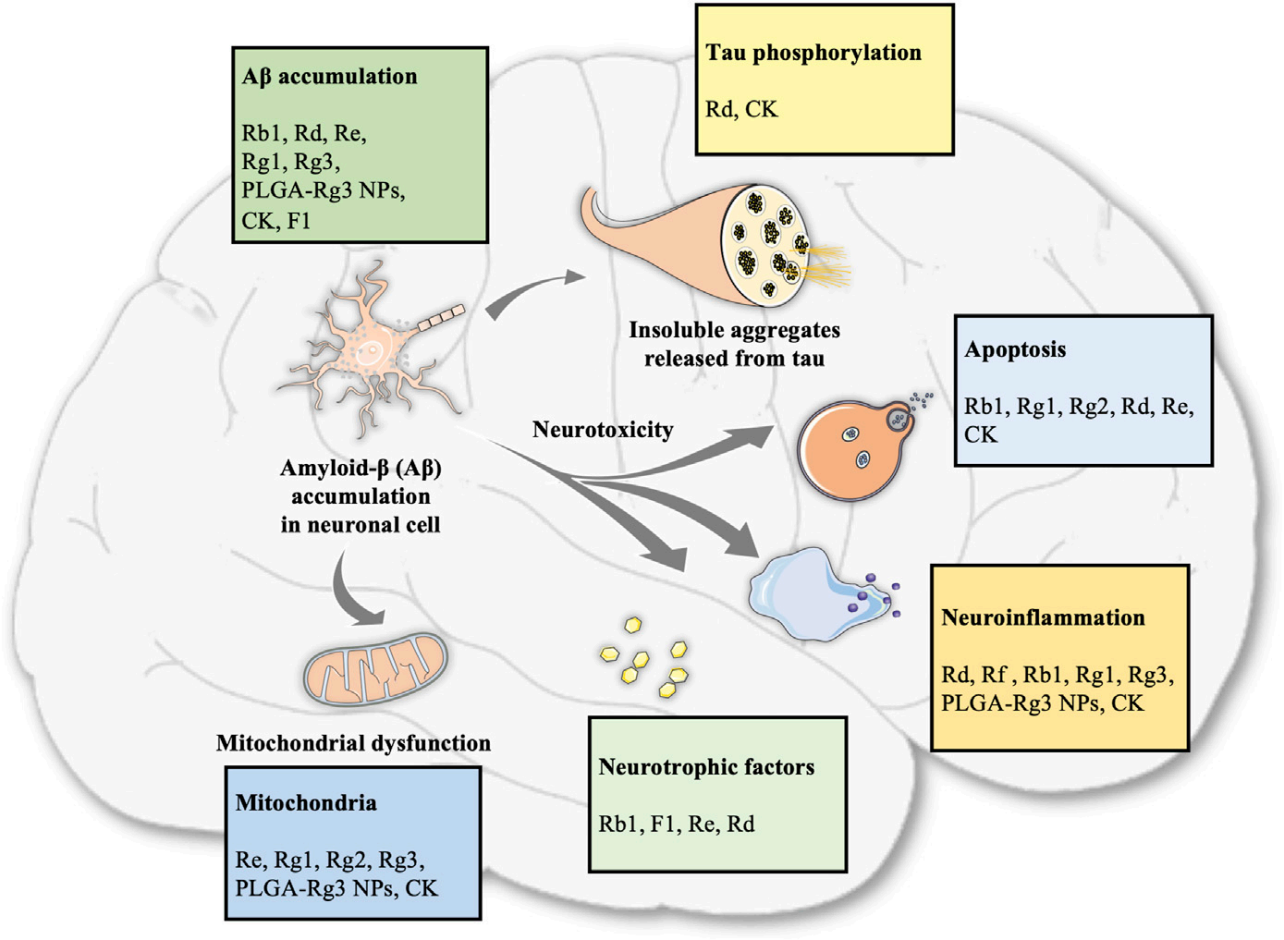
Summary of different ginsenoside monomers involved in various pathological processes of AD.

Overall, the neuroprotective effects of ginsenosides against AD are mediated by the regulation of Aβ accumulation, inflammation, apoptosis, neurotrophic factors, and mitochondrial function, as shown in Table 1 and Figure 1.

**TABLE 1:** Summary of effects and mechanisms of ginsenosides in neuronal cells and animal models.

Ref	Ginsenosides	Model	Inducer	Experimental model	Mechanism	Effects
Zhao et al. (2014)	Rg1	AD	Aβ_25–35_	NG108-15 neuroglial cells	TLR3, TLR4, NF-κB, TRAF-6, TNF-α, IFN-β, iNOS↓	Neuroinflammation
Li et al. (2019)	Rg1	AD		3 × Tg-AD mice	Arachidonic acid, 11b-PGF2a, cytc p450, enzyme prostaglandin-F synthase, tryptophan, lysine↓	Oxidative stress, inflammation reaction
Yang et al. (2020)	Rg1	AD		SAMP8 mice	Activated microglia cells, activated astrocytes, iNOS, Aβ↓	Oxidative stress, neuroinflammation
Xu et al. (2019)	Rg1	Neuronal damage	H_2_O_2_	Hippocampal neurons cells	β-Galactosidase, ROS, caspase-3, NOX2, p22phox, NLRP1, ASC, caspase-1, IL-1β, IL-18↓	Oxidative stress, apoptosis, neuroinflammation
Quan et al. (2020)	Rg1	AD	Aβ_1–42_	Rat hippocampal neurons cells	p-PPARγ, CDK5, BACE1, APP, Aβ1-42↓	Apoptosis, Aβ degradation and reduction
					IDE↑	
Nie et al. (2017)	Rg1	AD		3 × Tg-AD mice	SYN2, CPLX2, SNP25, PSD-95↑	Modulating the expression of the proteins of memory and depression
Cui et al. (2020)	Rg2	AD	Aβ_25–35_	Male SD rats	Caspase-3, Bax↓	Apoptosis
					Bcl-2, p-Akt↑	
Cui et al. (2017)	Rg2		Aβ_25–35_	PC12 cells	LDH, [Ca^2+^]i, ROS, caspase-3, Bax↓	Mitochondrial dysfunction, apoptosis
					p-Akt, Bcl-2↑	
Li et al. (2007)	Rg2		Glutamate	PC12 cells	[Ca^2+^]i, MDA, NO, calpain II, caspase-3↓	Anti-oxidation, anti-apoptosis
Joo et al. (2008)	Rg3	AD	Aβ_42_	BV-2 microglial cells	TNFα, IL-1β, iNOS↓	Neurotoxicity, microglial activation, inflammation
Lee et al. (2013)	Rg3	AD/learning and memory impairments	D-Galactose/LPS	Adult male SD rats	Caspase-3, caspase-9, Bax, AIF, cyto C, Bcl-2, TNF-α, IL-1β, COX-2↓	Mitochondrial dysfunction, energy metabolism, ETC, amino acid metabolism, purine metabolism, anti-apoptosis, neuroinflammation
Aalinkeel et al. (2018)	PLGA-Rg3 NPs	AD	Aβ_1–42_	C6/THP-1 cells	Cyto C, ROS, TNF-α, IL-1β↓	Aβ plaques, AβPP-A4, oxidative stress, mitochondrial dysfunction, neuroinflammation
Lin et al. (2019)	Rb1	AD	Aβ_1–40_	Male Wistar rats	IL-1β, Aβ, GFAP↓	Aβ plaques, neuroinflammation
Wang et al. (2018)	Rb1	AD	Aβ_1–40_	Male SD rats	Bax, caspase-3↓	Apoptosis
					Bcl-2↑	
Changhong et al. (2021)	Rb1	AD	Aβ_25–35_	PC12 cells	Cholesterol, ROS, lipid peroxidation↓	Apoptosis, PPARγ activation, cholesterol reduction
					PPARγ↑	
Zhao et al. (2018)	Rb1	AD	Aβ_1–40_	Male SD rats	Nestin, GFAP, NSE, NSCs, NPCs↑	Promote the proliferation and differentiation of endogenous NSCs
Yang et al. (2020)	Rg3 + Rb1	AD		SAMP8 mice	TNF-α, activated microglia cells, activated astrocytes, ASC, caspase-1, iNOS, Aβ↓	Oxidative stress, neuroinflammation
Han et al. (2019)	F1	AD		Old APP/PS1 mice	Aβ plaque↓	Amyloid protein (Aβ) accumulation
					pCREB, BDNF↑	
Du et al. (2018)	Rf	AD	Aβ_42_	N2A cells	ROS, Ca^2+^, IFN-γ↓	Apoptosis, neuroinflammation, oxidative stress
					Mmp, IL-13↑	
Yang et al. (2019b)	CK	Memory impaired	Scopolamine hydrobromide	ICR mice	SOD, GSH-PX, Bcl-2, IDE, Nrf2, HO-1↑	Aβ plaques, neurotoxicity, oxidative stress, apoptosis
					MDA, Bax, caspase-3, APP, BACE1, PS1, Aβ, Keap1↓	
Chen et al. (2019)	CK	AD	Aβ_1–42_	HT22 cells	IRS2, IDE, GLUT1, GLUT3↑	Aβ intake and accumulation, energy metabolism disorder
					GSK3β, tau↓	
Park et al. (2012)	CK	Inflammation	LPS	Male C57BL/6 mice/BV2 microglial cells/primary cultured microglia	Number of activated microglia, NO, TNF-α, IL-1β, iNOS, IL-6, MCP-1, MMP-3, MMP-9, ROS, NADPH, MAPKS↓	Microglial activation, NF-κB/ap-1 activities suppresses inflammatory molecules
					CREB↑	
Liu et al. (2019)	Re	AD	Aβ_25–35_	SH-SY5Y cells	Caspase-3/7, caspase-9, cyt C, ASK-1, JNK, Bax, ROS↓	Mitochondrial apoptosis, oxidative damage, oxidative stress
					Caspase-8, caspase-12→	
					MMP, ATP, Bcl-2/Bax, GSH, SOD, Gpx↑	
Cao et al. (2016)	Re	AD		N2A/APP695 cells	sAPPβ, C99, BACE1↓	Aβ production
					PPARγ protein and mRNA↑	
Li et al. (2018)	Re	AD	Aβ_25–35_	Male kunming mice	Tryptophan↓	Metabolomics
					LPC, hexadecasphinganine, phytosphingosine, phenylalanine↑	
Liu et al. (2012)	Rd	AD	Aβ_1–40_	Male SD rats	IL-1β, IL-6, TNF-α, S100β mRNA, PC, HNE, caspase-3↓	Inflammation, oxidative stress, apoptosis
					IL-10, HSP70 mRNA↑	
Liu et al. (2015a)	Rd	AD		APP transgenic mice	IL-1β, IL-6, TNF-α, S100β mRNA, NF-κB p65↓	Inflammation, NF-κB
					IL-10↑	
Li et al. (2013)	Rd	AD		APP transgenic mice	GSK-3β, Tyr216↓	p-tau
					Ser9, PP-2A↑	
Liu et al. (2015b)	Rd	AD	Aβ_25–35_	Primary cultured hippocampal neurons cells	ROS, Bax mRNA, caspase-3, cyto C mRNA↓	Oxidative stress, neuronal apoptosis
					SOD, GSH-Px, Bcl-2 mRNA↑	
Li et al. (2011)	Rd	AD	Okadaic acid	Adult male SD rats/Cortical neurons cells	Tau↓	Tau
					P-2A↑	
Yan et al. (2017)	Rd	AD	Ovariectomy/Inhibitor	Adult female rats/HT22 hippocampal neuronal cells	BACE1, Aβ↓	Activating estrogen-like activity
					sAPPα, ADAM↑	
Kim et al. (2014)	Re + rd			Neuro2a cells	ChAT, VAChT, ach, MAP-2, p75, p21, TrkA↑	Cholinergic markers

### Gintonin

The role of gintonin in the prevention and treatment of AD has been evaluated for many years. Gintonin exerts anti-AD effects by affecting Aβ plaque deposition, sAβPPα release, the cholinergic system, neurotrophic factors, autophagy and apoptosis, and G protein–coupled lysophosphatidic acid (LPA) receptors. Gintonin administration attenuates Aβ plaque deposition and stimulates sAβPPα release, improving memory impairment in mice with AD, suggesting that gintonin results in the formation of the beneficial sAβPPα rather than neurotoxic Aβ (Hwang et al., 2012). With respect to the cholinergic system, gintonin can increase choline acetyltransferase expression, causing the release of ACh and attenuating Aβ-induced cholinergic impairments in a transgenic AD mouse model (Kim et al., 2015a). The release and expression of the vascular endothelial growth factor (VEGF) in cortical astrocytes are stimulated by gintonin, which may be mediated by the LPA1/3 receptor or other receptors, exerting neuroprotective effects against hypoxia insults (Kim et al., 2016; Choi et al., 2019). Moreover, gintonin can induce autophagic flux in astrocytes *via* activation of the AMPK-mTOR signaling pathway and efficiently suppress the production of NO by regulating MAPK and NF-κB pathways (Saba et al., 2015; Rahman et al., 2020). Importantly, gintonin, an LPA receptor ligand, can interact with LPA receptors, which are abundantly expressed in astrocytes to induce transient increases in intracellular Ca^2+^ concentrations ([Ca^2+^]i), affecting neurotransmitter release and synaptic transmission and subsequently enhancing cognition. However, ginsenosides or other active components in ginseng have no effect on [Ca^2+^]i, which may be related to the chemical characteristics of gintonin and its action on G protein-coupled receptors (Im and Nah, 2013; Kim et al., 2015b; Choi et al., 2015).

### Other Extracts or Fractions of Ginseng

Apart from ginsenosides and gintonin, extracts or fractions from ginseng have also been widely investigated to explore their molecular mechanisms against AD in a series of cell and animal models. Ginseng extracts result in a reduction of Aβ amount, which may be related to multiple targets, including the balance between mitochondrial fusion and fission, basal respiration, and neuroinflammation attenuation in the AD brain (Chen et al., 2006; Shin et al., 2019). The Korean red ginseng extract, which may regulate alternative pathways such as mitochondrial dysfunction and Aβ degradation/clearance, inhibits tau aggregation but has no direct effect on Aβ_1–42_ accumulation (Shin et al., 2020). The oil from red ginseng, containing linoleic acid, β-sitosterol, and stigmasterol, exhibits protective effects against Aβ_25–35_-induced damage through inhibition of the NF-κB and MAPK pathway–mediated inflammation and apoptosis (Lee et al., 2018b). Red ginseng oil can also downregulate the p38/-JNK/-NF-κB pathway to suppress pro-inflammatory mediators, and caspase-3/PARP-1 signaling, inhibiting mitochondria-mediated apoptosis and protecting against Aβ-induced injury (Lee et al., 2017). In addition, the nonsaponin polysaccharide fraction, from ginseng, mitigates Aβ-induced neuronal dysfunction and improves mitochondrial respiration in the subiculum of the 5 × FAD mice model (Shin et al., 2021). Collectively, these results indicate that ginseng extracts and fractions have neuroprotective roles, improving mitochondrial dysfunction and inhibiting inflammation and apoptosis (Table 2). Importantly, the active components of these ginseng extracts should be further identified.

**TABLE 2:** Summary of effects and mechanisms of extracts or fractions from ginseng in neuronal cells and animal models.

Ref	Extract/fraction	Model	Inducer	Experimental model	Mechanisms	Effects
Lee et al. (2017)	Red ginseng oil	AD	Aβ_25–35_	PC12 cells	Ca^2+^, Bax, caspase-3, caspase-9, PARP-1, JNK, p38 NF-κB, iNOS, COX-2, PGE2, NO, TNF-α↓	Mitochondrial dysfunction, apoptosis, neuroinflammation
					MMP, Bcl-2↑	
Lee et al. (2018b)	Red ginseng oil	AD	Aβ_25–35_	PC12 cells	iNOS, p-NF-κB, COX-2, p-IκB, p38, p-ERK, p-JNK, Ca^2+^, Bax, caspase-8, caspase-9, caspase-3, RARP-1, TNF-α, IL-1β, NO, PGE2, iNOS, COX-2, p-p65↓	Oxidative stress, apoptotic responses, pro-inflammatory mediators
					MMP, Bcl-2↑	
Shin et al. (2021)	Nonsaponin fraction with rich polysaccharide (NFP) from red ginseng	AD	Aβ_1–42_	14 months old SD rats/5 × FAD mice/HT22 cells	Iba-1(+) area↓	Aβ deposition, neuroinflammation, neurodegeneration, mitochondrial dysfunction, impaired adult neurogenesis, cognitive dysfunction
					NeuN-positive cells, mitochondrial numbers, mitochondrial dynamics, OCR, ATP, mitochondrial respiration↑	
					Defective brain mitochondrial dynamics, number of DCX (+) neurons, dendritic morphology	
Shin et al. (2019)	KRG extracts	AD	Aβ_1–42_	5 × FAD mice/HT22 cells	4G8 (+) areaIba-1 (+), GFAP (+), Ki-67 (+),DCX (+)↓	Aβ accumulation, neuroinflammation, impaired adult neurogenesis, neuronal death, cognitive dysfunction, mitochondrial dysfunction
					Nonmitochondrial respiration↓	
					OCR, basal respiration, ATP-linked respiration Maximal respiration capacity↑	

### Formulas Containing Ginseng or Combination Treatment

**FIGURE 2:**
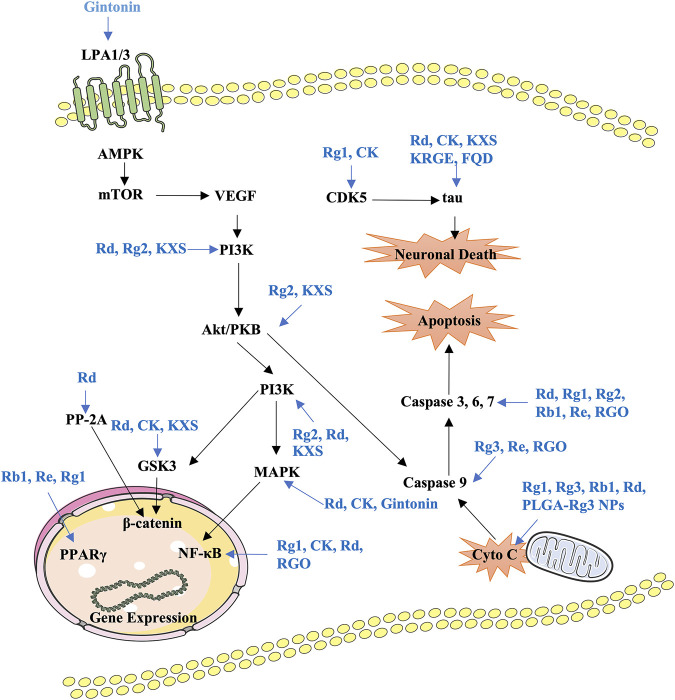
Summary of the functional effects of ginseng on AD *via* multiple links across regulatory mechanisms and multitarget effects. LPA, lysophosphatidic acid; KXS, Kaixin-San; PP-2A, protein phosphatase 2A; RGO, red Ginseng oil; Cyto C, cytochrome C.

Formulas containing ginseng and drug combinations can be used to achieve treatment efficacy and reduced toxicity. Currently, several decoctions containing ginseng are investigated to confirm the neuroprotective effects and identify the active components. Fuzheng Quxie decoction includes ginsenosides Rg1, Re, Rb1, and coptisine, which can cross the blood–brain barrier to inhibit tau hyperphosphorylation in the hippocampus, inhibiting learning and memory impairments in SAMP8 mice (Yang et al., 2017). The anti-neuroinflammatory effects of the Shenqi Yizhi formula in the 5 × FAD mice model may be mediated by active components including ginsenoside Rg1, astragaloside A, and baicalin by influencing energy metabolism, cytoskeleton, and stress reaction (An et al., 2018; Ren et al., 2020). Shengmai San can inhibit Aβ_1–42_ production to improve spatial learning and memory of APP/PS1 mice (Zhang et al., 2018). Kaixin San (KXS), a well-known formula that has been used in clinical settings for a long time, has various pharmacological effects; for instance, protecting nerve cells and preventing AD (Lv et al., 2014; Wang et al., 2019). The active components of KXS have been identified as ginsenoside Rf, ginsenoside F1, and dehydropachymic acid, which can activate cAMP-dependent signaling and promote neurotrophic factor synthesis in primary astrocytes and AD mice (Cao et al., 2018; Wang et al., 2019). Importantly, system biology analysis has validated that KXS has multitarget synergistic effects on the amelioration of AD features (Guo et al., 2019). Ninjin-yoei-to (NYT), a formula containing 14 herbs, can promote the production of nerve growth factor in rat embryo astrocytes after incubation for 24 h (Yabe et al., 2003). Additionally, GAPT (Jinsiwei), a combination of several active components, can reduce the AChE activity and expression and increase ACh synthesis to improve cholinergic nerve function, reducing the learning and memory impairments in scopolamine-induced mice (Liu et al., 2020). Pretreatment with *P. montana* and red ginseng extracts significantly reduces catalase and AChE activities, inhibiting trimethyltin-induced neuronal cell death, oxidative stress, and learning and memory impairments (Seo et al., 2018). Ginsenoside Rg1 combined with the Acori graminei rhizoma extract can reverse the effect of Aβ_1–42_ accumulation by regulating the expression of miR-873-5p in PC12 cells and SAMP8 mice (Shi et al., 2018). The current findings of formulas or combination treatment in AD have been summarized in Table 3. *In vitro* and *in vivo* preclinical studies have demonstrated that ginsenosides, gintonin, and other active components from ginseng or formulas containing ginseng mainly regulate PI3K/Akt, AMPK-mTOR, MAPK, GSK-3β/CDK5, NF-κB, and mitochondrial apoptotic signaling pathways to improve key pathological processes of AD development (Figure 2).

**TABLE 3:** Summary of effects and mechanisms of formulas containing ginseng in neuronal cells and animal models.

Ref	Formulas	Medicines	Model	Inducer	Experimental model	Mechanism	Effects
Yang et al. (2017)	Fuzheng Quxie Decoction	Renshen, huan glian, and chuanxiong (9:6:5)	AD		SAMP8 mice	p-tau↓	p-tau
						p-PP2A, NR2A, nissl bodies↑	
An et al. (2018)	SQYZ granules	Ginsenoside Rg1, astragaloside a, and baicalin	AD		APP/PS1 double transgenic mice	Aβ42, dynamin-1↓	Aβ deposition, neuroinflammation, stress responses, energy metabolism
						MAPK3, TCA (dalt, Fabp5, ldhb, Glo1, Eno1), HSP↑	
						Atp5b, Dmxl1	
Ren et al. (2020)	Shenqi yizhi granules	*Panax ginseng*, *Astragalus membranaceus*, and *Scutellaria baicalensis* Georgi (2:4:3)	AD		APP/PS1 double transgenic mice	Mdhc, PKM, ATP, HSP↑	Energy metabolism, stress response, cytoskeleton, synaptic transmission, signal transduction, amino acid metabolism
						acetyl-CoA	
Guo et al. (2019)	Kai-xin-san	*Panax ginseng*, *Polygala tenuifolia* Willd, *Poria cocos* (Schw.) Wolf, and *Acorus tatarinowii* Schott (3:2:3:2)	AD	Aβ_25–35_	SD rats/PC12 cells	AChE, Bcl-2, ROS, TNF-α, IL-1β↓	Oxidative stress, neuroinflammation, apoptosis, Aβ deposition, cytoskeleton
						Ach, Bax, cleaved-caspase-3, p-PI3K, *p*-Akt, and *p*-GSK-3β↑	
						PI3K/Akt, tau	
Cao et al. (2018)	Kai-xin-san	*Ginseng Radix et Rhizoma*, *Polygalae Radix*, *Acori Tatarinowii*, and *Poria* (3:2:2:3)			Primary mouse astrocytes cells	MMP-9, TIMP-1→	cAMP-dependent pathway, synthesis of neurotrophic factors *via* regulation of the tPA system
						NGF, BDNF, CREB, tPA↑	
Liu et al. (2020)	GAPT, GEPT, or jinsiwei	Ginseng, epimedium, polygala, and tuber curcumae	Learning and memory-disordered model	Scopolamine	6 months old male ICR mice	MDA, AChE, ROS↓	Protecting cholinergic neurons, reducing oxidative stress injury, neuroprotective
						ChAT, SOD, GPX, Ach↑	
Seo et al. (2018)	P*. montana* and red ginseng extracts	Hongshen and gegen	Neurodegeneration	TMT	5 weeks old male ICR mice	AChE↓	Ach, oxidative stresses
						Catalase, MDA↑	
Shi et al. (2018)	Rg1 and *Acori graminei Rhizoma*	Ginsenoside Rg1 and shichangpu	AD	Aβ_1–42_	SAMP8 mice/Primary hippocampal neurons cells/PC12 cells	HMOX1↓	Apoptosis
						mir-873-5p↑	

## Clinical Trials of Ginseng, Formulas, or Dietary Supplements Containing Ginseng

At present, very few clinical trials investigating the effects of ginseng intervention on AD are ongoing or completed. Most clinical trials focus on ginseng or red ginseng extract and employ the Alzheimer’s Disease Assessment Scale (ADAS) and the Mini-Mental State Examination (MMSE) scores to monitor cognitive performances. After ginseng treatment for 12 weeks, the cognitive subscale of ADAS and the MMSE score are significantly improved, indicating that ginseng has positive effect on the cognitive performance of AD patients (Lee et al., 2008). After administration with heat-processed ginseng (4.5 g/day) for 24 weeks, cognitive function and behavioral symptoms in patients with moderately severe AD are improved at 12 weeks, which is sustained for the next 12-week follow-up (Heo et al., 2012). AD patients in the high-dose (9 g/day) Korean red ginseng group show significant improvements on the ADAS and Clinical Dementia Rating scales after 12-week therapy compared with the control group (Heo et al., 2008). In a larger-sized study, oral administration of Memo^®^, a triple formula (750 mg lyophilized royal jelly, 120 mg *Ginkgo biloba* extract, and 150 mg ginseng extract) for 4 weeks was shown to exert beneficial effects on cognition during aging and pathologic cognitive impairment in the early stages of AD (Yakoot et al., 2013). Furthermore, a combination of NYT and donepezil is more effective for AD patients with mild depression compared with donepezil-only (Kudoh et al., 2016). In addition, no adverse reactions occurred in all clinical studies, which suggests that ginseng can be used safely and has better tolerance for the patients with AD. The findings from clinical trials have been summarized in Table 4, which preliminarily indicates that ginseng treatment is safe and has a positive effect on cognition in patients with AD. However, it is essential to conduct more and high-quality clinical trials to evaluate the protective and therapeutic effects of ginseng, formulas containing ginseng, and combinations with other drugs in patients with different stages of AD and explore the underlying molecular mechanisms.

**TABLE 4:** Summary of clinical trials of ginseng interventions in AD patients.

Ref	Medicine	Model	Sample size	Inclusion criteria	Evaluative criteria	Results
Lee et al. (2008)	Ginseng	AD	Control group (n = 39), ginseng group (n = 58)	1. NINDS-ADRDA	MMSE, ADAS	Ginseng as a cognitive enhancer for AD patients
				2. Patients without other neurodegenerative disorders or cognitive impairments		
				3. The use of drugs for concomitant conditions was permitted		
Kudoh et al. (2016)	Ninjin-yoei-to (renshen yangrong tang)	Mild to moderate probable AD	Donepezil (n = 11), donepezil + NYT (n = 12)	1. Patients diagnosed with AD between 65 and 85 years of age	MMSE, ADAS-J cog, NPI	No significant differences between the two groups
				2. Patients who scored 15–23 points on the MMSE after treatment with donepezil (5 mg/day) for more than 8 months, but who did not exhibit any significant change in cognitive function		
				3. Patients without an otherwise healthy condition		
Heo et al. (2012)	Heat-processed ginseng	AD	1.5 g/day (n = 10), 3 g/day (n = 10), 4.5 g/day (n = 10), control (n = 10)	1. Age older than 50 years	ADAS, MMSE	Significant improvement on the MMSE and ADAS. Higher dose group (4.5 g/day) showed improvements in ADAS and MMSE score as early as at 12 weeks, which sustained for 24-week follow-up
				2. MMSE score of ≤20		
				3. CDR score of ≥1		
				4. Without psychiatric disorder, seizure disorder, or a medical condition		
				5. Without cognitive impairment due to stroke, neoplasia, infection, hypoxic brain injury, or medications		
Heo et al. (2008)	Korean red ginseng	AD	Low-dose (4.5 g/day, n = 15), high-dose (9 g/day, n = 15), control (n = 31)	1. Aged older than 50 years and baseline MMSE score of≥10 and ≤26	ADAS, K-MMSE, CDR	High-dose KRG group was significant improvement on the ADAS and CDR but normally improved on the MMSE after 12 weeks of KRG therapy when compared with those in the control group
				2. Patients were without psychiatric disorder, seizure disorder, or a medical condition that would limit the completeness of the study		
				3. Patients without cognitive disorder caused by stroke, hypoxic brain, cerebral neoplasia, infection, and medications		
Yakoot et al. (2013)	Memo® (combining of lyophilized royal jelly, extracts of *G. biloba* and *P. ginseng*)	AD	Experimental group (n = 30) control group (n = 30)	1. Aged 50–80 years, complaining of memory impairment or forgetfulness	MMSE	Beneficial in treating the cognitive decline that occurs during the aging process as well as in the early stages of pathologic cognitive impairment of insidious-onset vascular dementia and in AD
				2. Satisfying the clinical criteria of memory complaint, normal activities of daily living, abnormal memory for age, and no documented dementia		

## Conclusion

**FIGURE 3:**
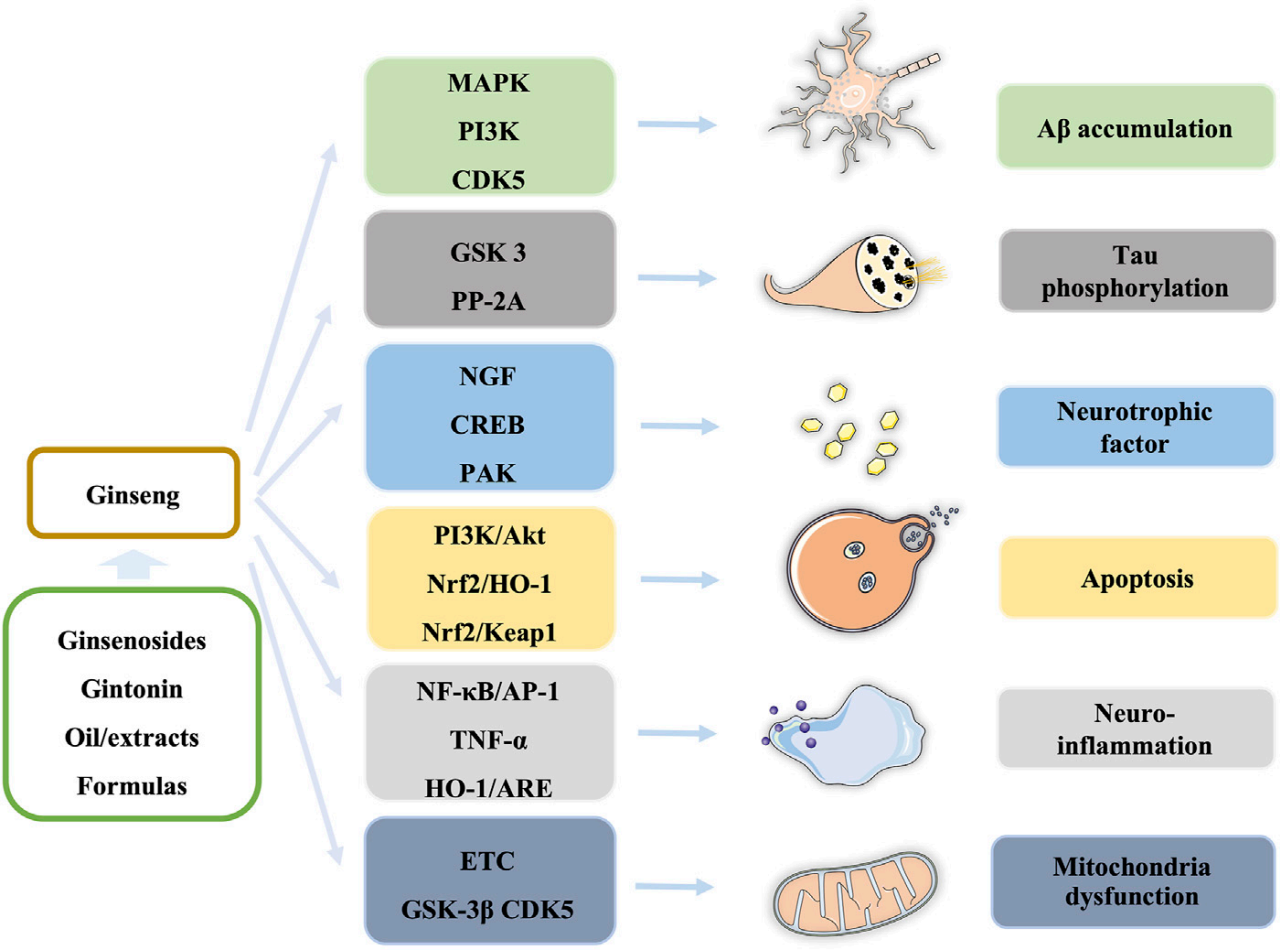
Summary of ginseng components and the affected pathways in the pathological process of AD.

In this review, we summarize our recent findings regarding the effects of ginseng on AD and cognitive and memory dysfunction. Ginsenosides, gintonin, extracts/fractions from ginseng, and formulas containing ginseng are widely studied in cells and animal models, which demonstrate that ginseng exerts neuroprotective effects in the prevention and treatment of AD through regulating multiple signaling pathways, such as PI3K/Akt, AMPK-mTOR, and NF-κB pathways, to block or improve pathological processes, including Aβ accumulation, tau phosphorylation, neuroinflammation, neurotrophic factors, apoptosis, and mitochondrial dysfunction in different stages of AD (Figure 3).

However, in preclinical and clinical studies of the effects of ginseng on AD, three important aspects should be considered: 1) Most studies focus on ginsenosides and gintonin with different chemical characteristics. The molecular mechanisms underlying the effects of ginsenosides and gintonin in the regulation of Aβ accumulation, neuroinflammation, and neurotrophic factors are similar, but only gintonin can interact with LPA receptors to mediate transient increases in [Ca^2+^]i regulating neurotransmitter release and improving cognition. 2) A series of cell models, such as PC12, SH-SY5Y, SweAPP-SK, and hippocampal neurons and several animal models, such as SAMP8, 5 × FAD, and 3 × Tg-AD mice are used to evaluate the neuroprotective effects of ginseng. Based on the current preclinical findings, we think that long-term interventions with ginseng or its formulas are critical to improve cognitive features for AD patients in early stages, which should be validated in larger and multicenter clinical trials. 3) Key pathological procedures of AD, including Aβ synthesis and degradation, neurotoxicity, and mitochondrial function, are potential targets for ginseng treatments. However, the molecular targets and binding sites of ginsenosides, gintonin, and other components in the prevention and treatment of AD remain unclear. Therefore, the network of targets of ginseng needs to be explored in the future. Collectively, this review can provide new insights into the possible use of ginseng in the prevention and treatment of AD.
